# Editorial: Novel insights into aortic arch repair

**DOI:** 10.3389/fcvm.2022.1087952

**Published:** 2022-12-13

**Authors:** Massimo Bonacchi, Francesco Cabrucci, Beatrice Bacchi, Maruti Haranal, Sandro Gelsomino, Basel Ramlawi, Aleksander Dokollari

**Affiliations:** ^1^Cardiac Surgery F.U., Experimental and Clinical Medicine Department, University of Florence, Florence, Italy; ^2^Department of Cardiac Surgery, U. N. Mehta Institute of Cardiology and Research, Ahmedabad, India; ^3^Department of Cardiac Surgery, Carom School for Cardiovascular Diseases, Maastricht University, Maastricht, Netherlands; ^4^Department of Cardiac Surgery Research, Lankenau Institute for Medical Research, Main Line Health, Wynnewood, PA, United States

**Keywords:** aortic arch repair, aortic arch advancement, thoracic endovascular aortic repair (TEVAR), frozen elephant trunk (FET), new strategy, thoracic aortic aneurysm and dissection (TAAD)

Since the pioneering work of DeBakey and Cooley 60 years ago, aortic arch repair has been always considered a challenge for cardiac surgeons.

On one side, it can be considered a nightmare due to its invasiveness, technical complexity, highly demanding procedures, and high mortality and morbidity rates. On the other side, the desire to be capable of treating such complex conditions pushes forward the boundaries of surgery and endovascular procedure, therefore, leading to new treatment methods attracting also innovative technologies. For decades, surgeons worked in a dichotomy fashion: TAAD vs. TBAD, surgery vs. medical management, and TAR vs. a more conventional approach. The advent of TEVAR launched a new era in the thoracic aorta and aortic arch treatments. Now more than ever, cardiovascular surgery feels the need to set a balance between traditional open surgery, hybrid, and endovascular procedures to improve outcomes and rise the standard of care once again. The articles featured in this collection attempt to address some of these important topics and provide readers with new insights into current and emerging surgical techniques for aortic arch repair surgery.

Endovascular solutions unleashed plenty of innovative and combined strategies forcing surgeons to create new *ad hoc* classifications and terminology to analyze the outcomes. The global adoption of Ishimaru's “anatomical endograft zone map” (1) proposed in early 2000 and led by Bavaria et al. (2) and Hughes et al. (3) created and expanded a classification by sorting HAR into “zones” and “types.” However, a large gap in the literature still exists regarding ATAAD. Responding to this void, Qiu et al. showed that the Fuwai classification, where the proximal aortic arch is the proximal end of the innominate artery and includes the proximal part of the left common carotid artery, while the distal aortic arch is the proximal end of the left common carotid artery and includes the distal end of the left subclavian artery, fully responds to the modern era classification demands and can work also for acute management.

Nowadays, aortic arch surgery is at a crossroads. In one direction, the largest volume centers in Asia have been demonstrating that is possible to treat the whole spectrum of arch pathology in a totally endovascular fashion thanks to the new “anti-type I endoleak weapons.” Bao et al. were able to treat 12 patients with ruptured aortic arch lesions with a chimney graft technique (50% received triple chimney), and despite the high burden of post-operative type I endoleak (58%) they successfully re- treated patients using coil and Onyx glue to fill the gutter, therefore, reaching an astonishing 100% overall technical success. Moreover, Li, Shu, Li et al. proposed their “self-radiopaque marker guiding physician-modified fenestration” or “S-Fenestration” technique as means to break down the catastrophic event of misalignment during PMF TEVAR. The S-Fenestration technique reaches an instant success rate of 98.4% with a similar branch patent artery rate but it should be noted that only 2 out of 140 treated patients received fenestrations for all three arch vessels (Li, Shu, Li et al.). Besides, Li, Shu, Wang et al. pioneered the first-in-human implantation of the gutter-free stent graft for *in situ* fenestration TEVAR. This new gutter-free C-skirt stent promises to eliminate endoleak and creates a strong fixation between the branch and the aorta endograft pieces (Li, Shu, Wang et al.).

On the other direction, it exists a “real world” aortic arch surgery made of emergent procedures, complex anatomy, lack of hybrid operative room, redo intervention, congenital conditions, and collagenopathies to deal with (Carrel et al.) (4, 5). In this context, Tirone-David expresses concerns that aortic arch replacement is not appropriate for the “general cardiac surgeon” (6). On the other hand, it has to be taken into account that Sa et al.s' meta-analysis demonstrated that an aggressive approach seems to confer better long-term survival and lower risk for reoperation in the follow-up of patients treated for ATAAD (7). Following these considerations, many new surgical techniques, perfusion strategies, and graft devices have been introduced in the last few years: from ABO perfusion strategy to normothermic frozen elephant trunk surgery without circulatory arrest, and from new FET graft devices to different types of HAR. In this context, Xie et al. demonstrated that among 633 patients with type I aortic dissection, total arch replacement with traditional technique, total arch replacement with FET, and ABO techniques had comparable clinical outcomes and overall mortality of 6.6%. It is important to mention that the ABO technique may need further investigation to show its potential (Xie et al.). Finally, Singh et al. in their comprehensive review underlined how the real-life practice of cardiac surgeons tends to be an open approach for emergent situations and different types of HAR depending on the anatomy and expertise of the center for chronic aneurysms (8, 9).

In conclusion, the vast amount of armamentarium for the treatment of aortic arch pathologies has led us to carefully consider the indications for one procedure vs. the other based on anatomy, age, urgency, STS score, and team-based decision. In this context, improved outcomes can relate to two main considerations; (1) the referral center should have a dedicated experienced team of cardiac surgeons and vascular surgeons that provide patient-tailored treatment, (2) the center must be a high-volume center with experience in combining surgical and endovascular approaches.

To obtain optimal results it is necessary, in our opinion, to improve the clinical care strategy of patients who undergo Aortic arch repair. To achieve this goal, a multidisciplinary approach to the patient with aortic arch pathology is mandatory, ideally a real “Aortic Team” with close collaboration among various professionals and specialists to improve all phases of the clinical path: from diagnosis and definition of anatomical aspects, from the design to the production of more tailored, personalized and biocompatible devices, to pre-implant evaluation systems, from postoperative management to the reintegration of the patient into an active and productive life.

In summary, this innovative and advanced approach in the context of the “new era” in the treatment of aortic arch repair, is probably the most important key factor toward full implementation and success for such a challenging and fascinating new therapeutic frontier.

## Author contributions

MB, FC, BB, MH, SG, BR, and AD contributed to conception and design of the study. MB, FC, and AD wrote the first draft of the manuscript. MB, FC, BB, and AD wrote the second draft of the manuscript. All authors contributed to manuscript revision, read, and approved the submitted version.
